# Iron Supplementation of Pregnant Sows to Prevent Iron Deficiency Anemia in Piglets: A Procedure of Questionable Effectiveness

**DOI:** 10.3390/ijms25074106

**Published:** 2024-04-08

**Authors:** Rafał Mazgaj, Paweł Lipiński, Rafał R. Starzyński

**Affiliations:** 1 Department of Molecular Biology, Institute of Genetics and Animal Biotechnology Polish Academy of Sciences, 05-552 Magdalenka, Poland; r.mazgaj@igbzpan.pl; 2 Laboratory of Metalloprotein Biology, Institute of Biochemistry and Biophysics Polish Academy of Sciences, 02-106 Warsaw, Poland

**Keywords:** sows, iron metabolism, piglets, anemia, iron supplementation, pig

## Abstract

In pigs, iron deficiency anemia (IDA) is a common disorder that occurs during the early postnatal period, leading to the stunted growth and increased mortality of piglets. The main cause of IDA is low iron stores in the liver of newborn piglets; these stores constitute the main source of iron needed to satisfy the erythropoietic requirements of the piglets in their first weeks of life. Insufficient iron stores in piglets are usually due to the inadequate placental iron transfer from the sow to the fetuses. Therefore, iron supplementation in pregnant sows has been implemented to enhance placental iron transfer and increase iron accumulation in the liver of the fetuses. Over the years, several oral and parenteral approaches have been attempted to supplement sows with various iron preparations, and consequently, to improve piglets’ red blood cell indices. However, there is debate with regard to the effectiveness of iron supplementation in pregnant sows for preventing IDA in newborn piglets. Importantly, this procedure should be carried out with caution to avoid iron over-supplementation, which can lead to iron toxicity. This article aims to critically review and evaluate the use of iron supplementation in pregnant sows as a procedure for preventing IDA in piglets.

## 1. Introduction

In domestic pigs (*Sus scrofa domestica*), neonatal iron deficiency anemia (IDA) occurs in almost all contemporary breeds [1,2]. Iron deficiency is a long-standing problem in the pig farming industry, with the first case of this disorder reported in the late 19th century [3]. Then, in 1924, a causal link was made between iron deficiency and anemia [4]. In newborn piglets, IDA has since been an ongoing problem in pig rearing, and many studies, including our own, have been dedicated to various aspects of this pathology [1,2,3,5,6]. In suckling piglets, IDA is typically a hypochromic, microcytic anemia, characterized by decreased red blood cell (RBC) indices. Without iron supplementation, piglets rapidly develop IDA, and thus, by definition, the values of RBC parameters measured in these animals are below acceptable ranges. There is a general consensus that the hemoglobin concentration cut-off level for anemia in suckling piglets is 8 g/dL [7]. To prevent IDA in young piglets, supplemental iron from an exogenous source must be administered during their first days of life. Parenteral iron supplementation of piglets is usually carried out by a single intramuscular injection of 200 mg of iron dextran complex within 2–3 days after birth. This method of supplementation is routinely used in pig farming and is considered the gold standard in IDA prevention in piglets [8]. However, a single high dose of iron administered parenterally may disturb the fragile iron homeostasis of newborn piglets. Providing large amounts of iron to piglets substantially increases the expression of hepcidin—a key systemic regulator of iron metabolism [9], and consequently, via function of the hepcidin–ferroportin regulatory axis [10], may inhibit the release of iron to the blood from enterocytes, macrophages, and hepatocytes. Parenteral iron administration can also lead to more dramatic outcomes, such as sudden cardiovascular collapse and respiratory failure [11].

The need for early postnatal supplementation is due to the low fetal iron reserves in newborn piglets (they are born with 50–70 mg of iron in the liver) [1]. The hepatic iron content in piglets at birth covers their iron requirement for only the first 3–4 days of postnatal life. Indeed, in piglets not supplemented with iron, the hepatic iron content is five times lower on day 4 after birth compared to day 1 after birth, and on day 7, it is barely detectable [12]. Moreover, the iron content in sow’s milk has been reported to range from 1.4 to 2.6 µg/mL [13], which is not sufficient for the development of piglets. Notably, the daily requirement of iron for fast-growing piglets in the first weeks of life is approximately 7–18 mg of iron [1]. The low hepatic iron content of piglets at birth is possibly an outcome of increased litter sizes and high birth weights, which are two objectives of the selective breeding program of contemporary pigs. Consequently, the iron transferred from the pregnant sow must be distributed among a greater number of fetuses with greater birth weight. Mahan and Newton [14] found that highly prolific sows have lower body iron content than sows with decreased prolificacy. Moreover, highly prolific sows may provide less iron to the developing piglets [15]. In contrast, our recent study showed that RBC and iron status (including hepatic iron content) in newborn piglets are not primarily determined by the litter size [16]. Nevertheless, the main objective of iron supplementation in pregnant sows is to increase their iron levels, and in turn, to provide their fetuses with the extra iron required to meet the erythropoietic needs of newborn piglets. This review focuses on the history and results of supplementing sows with iron during pregnancy to enhance the iron transfer to the fetuses and increase their iron stores.

## 2. The Placenta: The Unappreciated Organ of Iron Metabolism

**Figure 1:**
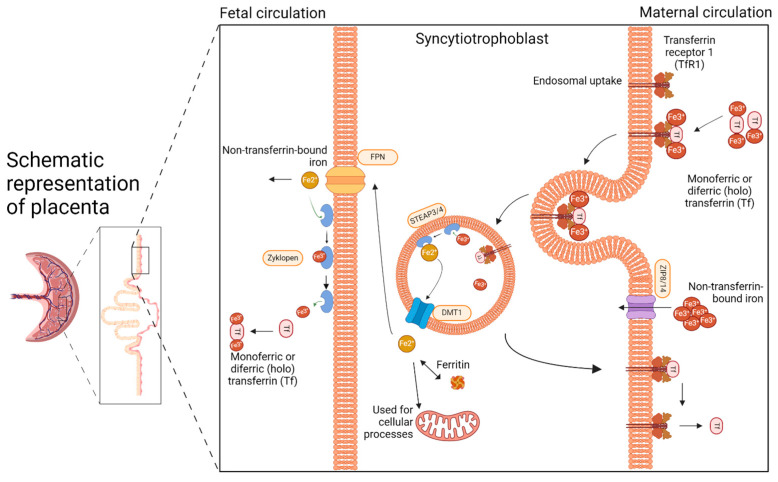
Pathways of iron transfer across the placenta. Created with BioRender.com.

Knowledge about iron transport across the placenta is crucial for planning iron supplementation in pregnant sows. In 2016, after Cao and Fleming [17] recalled the importance of the placenta in iron metabolism, much research has been devoted to transplacental iron transport and its regulation [18,19]. The placenta is a highly vascularized organ responsible for the exchange of nutrients and gases between the mother and developing fetus. The porcine placenta is classified as diffuse epitheliochorial [20]. This designation distinguishes it from the placentae of other livestock species in that there are no placentomes (making it diffuse), and both the fetal and maternal epithelial cell layers are maintained throughout gestation (making it epitheliochorial). Sows are pregnant for approximately 115 days, with a normal pregnancy duration ranging from 111 to 120 days depending on the breed. In pigs, fetal iron stores are generated in the liver during the 10 weeks before birth [21]. This period is critical for building up the fetal iron stores that are used in the first weeks of postnatal life (before piglets can absorb sufficient amounts of iron from the feed [22]) to support their rapid growth and erythropoiesis. Several mechanisms improve iron availability in the maternal circulation during pregnancy, including both increased intestinal iron absorption and the release from maternal iron stores [21]. As pregnancy progresses, maternal absorption of non-heme iron increases, and heme iron absorption likely follows a similar trend [21,22]. Maternal iron stores are mobilized from the liver and spleen during pregnancy, as shown in studies of pregnant pigs [23] and rodents [24,25]. Hepcidin is a small peptide hormone produced mainly by hepatocytes that orchestrates body iron fluxes (including iron transfer across the placenta) by adjusting iron supply body iron requirements. Hepcidin binds to ferroportin (Fpn) to induce its degradation, thus inhibiting iron release from exporting cells [23]. Its expression is controlled by iron levels, erythropoietic activity, and inflammatory cues [24,25]. In pregnancy, maternal and fetal hepcidin is regulated by both maternal and fetal iron conditions [26]. Iron supplementation of sows during pregnancy is intended to increase maternal iron status, and consequently, to enhance the efficiency of iron transfer to the developing fetuses. That is why understanding the mechanisms of placental iron transport is crucial to properly conduct this procedure. The main pathways of iron transport across the placenta are depicted in Figure 1. 

Iron is delivered to the placenta by the maternal circulation, where it is mainly bound to transferrin (Tf), forming a monomeric or diferric (holo) Tf-Fe^3+^ complex. The primary route of iron uptake by the placenta involves the uptake of Tf-bound iron from the maternal circulation through transferrin receptor 1 (TfR1) on the apical membrane of the placental syncytiotrophoblast facing the maternal circulation [17,27]. The Tf-Fe^3+^ complex binds to TfR1 and then is internalized into the cell by clathrin-mediated endocytosis. In the acidic environment of the endosome, ferric ions dissociate from Tf and are then reduced to the ferrous (Fe^2+^) state by ferrireductases, possibly by STEAP3 (six-transmembrane epithelial of prostate) or STEAP4 [28]. Following the release of iron, the apo-Tf-TfR1 complex is recycled back to the membrane. The neutral pH of the extracellular space facilitates the dissociation of apo-Tf from TfR1 and its release back to the maternal circulation. TfR1 is then again available for the uptake of the Tf iron complex. Within the syncytiotrophoblast, ferrous iron is transported out of the endosome into the cytoplasm by divalent metal transporter 1 (DMT1) [29,30]. Studies in knock-out DMT1 mice clearly indicate that DMT1 is dispensable for iron transport across the placenta [31]. Other potential iron transporters expressed in the placenta include the Zrt/Irt-like proteins ZIP8 and ZIP14, members of the solute carrier family 39A (SLC39A) [32]. ZIP14 has been shown to transport non-transferrin-bound iron [33]. Similarly to DMT1, ZIP14 seems to be dispensable in the placental iron transport process, as null ZIP14 mice have normal iron stores [34]. In contrast, ZIP8 deficiency was found to cause anemia and lethality in mouse embryos [35], which demonstrates that ZIP8 is essential for prenatal development. Once in the cytoplasm, iron can be stored as ferritin (Ft), used for cellular processes, or exported to the fetal circulation via Fpn, the only known mammalian iron exporter. In this process, Fpn cooperates with zyklopen, a copper-dependent ferroxidase [36], which oxidases ferrous to ferric iron that can be bound by fetal Tf. Zyklopen is not essential for iron transport to the fetus in mice [37]. 

## 3. Supplementation of Pregnant Sows with Iron

### 3.1. Oral vs. Parenteral Iron Supplementation

In the literature, two main ways of administering iron to sows have been described: oral supplementation, in which iron is added to feed, or parenteral supplementation, in which iron compounds are administered via intramuscular injection. 

#### 3.1.1. Oral Iron Supplementation

Oral iron supplementation was the first procedure used to treat iron deficiency in piglets by increasing iron in pregnant sows. In one of the first studies to report IDA in piglets, McGowan and Crichton [4] attempted to prevent anemia in piglets by oral supplementation of sows during pregnancy. Subsequent studies by Hart [38] and Hooks [39] also addressed this issue. Oral iron supplementation of sows is a widely employed approach in pig farming. However, it is essential to consider both the positive and negative aspects associated with this procedure. 

In terms of the positive aspects of oral supplementation, ease of administration is an obvious one, as iron can be provided to sows simply by adding iron formulation to their feed [40]. Moreover, this type of iron supplementation does not cause distress to the sows in a critical condition, such as pregnancy [41]. However, iron absorption from the gastrointestinal tract can vary depending on the iron formulation used, the sow’s physiology and gut health, the concurrent dietary intake, or both components. This variability in absorption may result in inconsistent iron availability and effectiveness.

High oral iron doses or inappropriate supplementation protocols can also disrupt the gut microbiota in pregnant sows, potentially leading to dysbiosis and associated gastrointestinal disorders [42]. Maintaining gut health is crucial for optimal nutrient utilization and the well-being of both the mother and developing piglets. Excessive oral iron supplementation can lead to iron overload in pregnant sows, causing oxidative stress and tissue damage [43]. Iron excess can also predispose sows to infections and negatively impact various organs, including the liver and heart [12]. Proper dosing and monitoring of supplemental iron are necessary to prevent iron overload and its associated risks. Determining the optimal timing and duration of oral iron supplementation during pregnancy is essential. Administering iron during critical stages, such as early to mid-gestation, should support maternal iron stores and promote optimal piglet development. However, prolonged supplementation beyond the necessary period should be avoided to minimize the risk of iron overload in the sow.

#### 3.1.2. Parenteral Iron Supplementation

Parenteral intramuscular iron supplementation is a commonly employed method to alleviate iron deficiency in piglets; only a few studies have implemented this method of iron supplementation in pregnant sows [44,45,46,47]. It is essential to consider both the benefits and risks associated with this supplementation approach.

Parenteral intramuscular iron injection effectively and rapidly improves iron levels in pregnant sows, addressing iron deficiency and preventing anemia [44,45]. Improving iron levels leads to enhanced oxygen transport, energy metabolism, and overall health during pregnancy. Adequate iron applied through parenteral intramuscular supplementation positively impacts reproductive performance in pregnant sows. Improved iron levels promote better fertility, larger litter sizes, and higher birth weights, leading to healthier piglet outcomes; however, the effect of this form of supplementation on improving the level of liver iron reserves in newborn piglets is negligible [44]. Thus, parenteral supplementation of iron is limited only to preventing anemia in pregnant sows. In terms of the risks, parenteral intramuscular iron injections may cause local site reactions, such as pain, swelling, or abscess formation. Additionally, it can cause systemic iron overload [12].

The most common method of iron supplementation in pregnant sows, as described in this review, is the addition of iron formulation to the sow’s fodder during pregnancy. This choice is mostly dictated by the ease of the approach and the fact that veterinary staff are not needed, unlike with parenteral iron supplementation.

### 3.2. Doses and Formulations of Supplemental Iron

Doses of supplemental iron described in research articles vary from low to very high amounts (milligrams to grams, respectively) given orally or intramuscularly. Table 1 presents an overview of iron formulations, doses, and corresponding effects. It is worth mentioning that the dose of the supplementation also determines the form of iron administration. Excessive amounts of iron cannot be administered parenterally because of iron toxicity. 

The simplest formulation broadly used in the pig farming industry is orally supplemented inorganic iron in the form of ferrous oxide [4] or ferrous sulfate [48,49]. Ferrous oxide, a compound that contains iron in the +2 oxidation state (Fe^2+^), is used to provide elemental iron to the body. Ferrous sulfate, an iron salt that also contains Fe^2+^, is often given as either ferrous sulfate monohydrate (containing ~32% elemental iron) or ferrous sulfate heptahydrate (~20% elemental iron). Ferrous sulfate is more widely used than ferrous oxide because it has better bioavailability and is relatively inexpensive. The dose of iron in its inorganic form varies from 100 ppm per day to 700 ppm per day [48,49] and is given daily with feed. This inorganic iron formulation is currently the most used form of oral supplementation in pig farming [40]. 

Another form of dietary supplemental iron used in pregnant sows is the chelated form of iron, mostly as a complex with amino acids [50]. Chelation is a chemical process where metal ions, such as iron, are bound to organic molecules like amino acids to create stable complexes. The resulting chelated iron is better absorbed than non-chelated iron. Amino acid chelated iron has been formulated to increase the bioavailability of iron. Some commonly used amino acids for iron chelation include glycine, lysine, and methionine. These amino acids are known to form stable complexes with iron, improving its solubility and stability in the gastrointestinal tract and facilitating its absorption [51]. Chelated iron is thought to be transferred across the intestinal barrier by routes different from those by which inorganic iron is transferred: the transfer uses amino acid transporters, thus bypassing the canonical route of iron transporters. This form is expected to allow iron to enter the circulation using an alternative pathway, which avoids tightly regulated canonical pathways of iron absorption employing iron transporters controlled by hepcidin, iron regulatory proteins, or hypoxia inducible factor 2. 

The daily dose of amino acid chelated iron varies from 300 mg to 651 mg [50,52]. Formulations used in this type of supplementation are ferrous fumarate, iron lactate, iron glycine chelate, iron methionine chelate [50], and ferrous N-carbamylglycinate [53]. These forms of iron supplementation have been shown to have a positive influence on the hematological status of newborn piglets [15,50,52]. 

Lactoferrin [53,54,55], a milk protein with a high affinity for iron, is a promising carrier for delivering iron across the intestine to the circulation. Its ability to bind and transport iron in a non-toxic manner reduces the risk of iron-induced oxidative stress, which is often associated with traditional iron supplementation. By acting as a shuttle for iron, lactoferrin can facilitate its controlled and targeted release, potentially improving bioavailability and reducing the risk of iron overload [56]. Daily doses of lactoferrin-bound iron vary from 200 mg/kg of feed to 1 g/kg of feed. However, supplementing sows with lactoferrin showed no improvement in terms of the piglets’ hematological status [56]. Moreover, the lactoferrin–iron complex as an iron supplementation formulation is more expensive than simple inorganic formulations.

With new technologies, it has been possible to develop new, more sophisticated oral iron formulations. Novel sucrosomial ferric pyrophosphate (SFP) can improve iron absorption while reducing gastrointestinal side effects. It consists of tiny particles of ferric pyrophosphate encapsulated in a sucrosomial matrix. The sucrosomial matrix helps to protect the iron from interacting with the acidic environment in the stomach, and thus reduces gastrointestinal irritation [57]. The main postulated benefit of sucrosomial ferric pyrophosphate is enhanced iron absorption. The sucrosomial matrix allows the iron to be absorbed more efficiently in the small intestine, bypassing canonical routes of iron absorption. It is postulated that this form of encapsulated iron is taken up by intestinal cells as a whole complex, without the mediation of specific iron transporters [58]. In our previous research, we gave 60 mg of Fe per sow per day [23] and found that SFP did not significantly affect the systemic iron homeostasis of sows during pregnancy or the hepatic iron stores of the newborn piglets. We hypothesized that additional iron administered orally to healthy pregnant sows is insufficiently transferred across the placenta [59].

Another form of oral iron supplementation is the organic form of iron in the heme complex [58]. Heme, a ferrous iron protoporphyrin IX complex, is engaged as a prosthetic group in a number of heme proteins (including cytochromes, hemoglobin, and myoglobin) that is involved in important cellular and systemic physiological processes. Heme iron is highly bioavailable, as up to 30% can be absorbed, whereas the absorption of non-heme iron is more variable (1–10%) [60]. The absorption of heme iron occurs through specific heme transporters present in the small intestine, the main one being heme carrier protein 1 (HCP1). HCP1 imports heme iron into the enterocytes, where heme molecules are degraded by heme oxygenase 1 to yield ferrous iron, which can consequently be exported from enterocytes into the bloodstream via Fpn [61]. Later, it was shown that HCP1 has high affinity for folates, and its loss of function causes folate malabsorption [62]. Another candidate for intestinal heme absorption is the heme-responsive gene, expressed in the small intestine, where it can function as a heme importer [60]. Heme iron as a supplement looks promising, but apart from the high cost of such an iron preparation, experimental studies have yielded inconclusive results as to its effectiveness [6,63]. In our studies, we administered heme iron to pregnant sows and found no effect on the fetuses or newborn piglets (unpublished data). 

The intramuscular iron supplementation of pregnant sows uses only one iron formulation, iron dextran (FeDex), which is widely used in the pig farming industry. Iron dextran is a complex of iron and the carbohydrate dextran. The structure of FeDex consists of iron compound (usually in the form of ferric hydroxide) molecules bound to dextran, a starch-derived polysaccharide [64,65]. When FeDex is administered intramuscularly, it is released slowly into the circulation; the iron is then taken up from plasma by the macrophages (called Kupffer cells) of the reticuloendothelial system of the liver, spleen, and bone marrow, where iron is released from the iron–carbohydrate complex to join a cellular low-molecular-weight iron pool. Iron is then either stored in ferritin, the major tissue iron-storage protein, or released from the cell bound to Tf, to circulate in plasma and be distributed to tissues, particularly the bone marrow [66]. In reports focusing on iron supplementation in pregnant sows, the doses used in the experimental designs ranged from 22 mg to 300 mg of Fe [44,45,47], usually given in a single injection, but Ducsey et al. [47] describe a supplementation regimen in which single dose of FeDex is divided into five smaller doses and injected on days 40, 45, 50, 55, and 60 of pregnancy. This administration aimed to avoid the negative effects of FeDex intramuscular injections, though the authors concluded that treating the sow would not likely eliminate the need for FeDex injection in piglets to prevent anemia. Current studies and newborn piglet rearing practices clearly show that FeDex given to newborn piglets by intramuscular injection can prevent them from developing IDA, but there is no clear evidence that the same treatment in sows during pregnancy can increase prenatal iron stores in fetuses through placental transfer [8,12]. 

All of the forms of iron supplementation mentioned above are given in addition to the standard diet for pregnant sows. The first official nutrient recommendations for swine were published in 1944 by The National Research Council, long after the first reported cases of IDA in pigs [67]. Since then, there have been 11 updates, each of which eliminated requirements and recommendations for various nutrients that were no longer relevant or appropriate and added new ones. In the 11th Edition of the Nutrient Requirements of Swine, the recommended daily amount of iron for gestating sows is 168 mg per day [40]. However, it is important to mention that in most studies, iron preparations were given in addition to the standard iron supplement in sow feed, which consisted mainly of corn. The earliest studies reported only the composition of the feed, but not the basic iron content. In later studies, the initial iron content in the diet of pregnant sows began to be reported, and ferrous sulfate was the iron preparation used due to its cost-effectiveness. In peer-reviewed studies, the dietary iron content ranged from 20 mg/kg [68] of feed to 190 mg/kg [68] of feed. The iron content of the feed was usually calculated [68], or a standard commercial feed was used and the iron contents were provided by the supplier [54]. 

### 3.3. The Iron Levels of Pregnant Sows and Their Impact on Piglets

If left untreated, naturally developing IDA in piglets causes enormous economic losses due to slower growth rates and, more importantly, mortality [1]. All of the abovementioned iron formulations were intended to improve the iron status of pregnant sows, but more importantly, they were designed to enhance the transport of this microelement to developing fetuses and create an iron reserve in the fetal liver large enough to be sufficient for the first few weeks of the piglets’ life [57]. It was assumed that improving the iron status of pregnant sows would benefit that of the offspring, thereby eliminating the need for postnatally supplementing piglets with iron during their first days of life.

In early research, Pilgrim and Quershi [59] observed that piglets born to sows supplemented with iron had higher hemoglobin levels and better growth rates than piglets born to non-supplemented sows. They found that the iron status of the supplemented sows was significantly improved compared to females from the control group. Pregnancy has a significant impact on the fitness of the sows, as reported by Pond et al. [61]. Hemoglobin levels in pregnant sows at the end of pregnancy are significantly lower if females are not supplemented with iron. O’Connor et al. [69] also noted that during pregnancy, sows that were supplemented with iron in their feed did not experience a drop in hematological parameters at the end of pregnancy. These observations suggest the need to supplement pregnant sows not only to prevent the development of IDA during pregnancy but also to possibly increase iron transport to the developing fetuses. 

In some of the reviewed studies, iron supplementation was provided throughout pregnancy, while in others, supplementation was provided only during the last trimester of pregnancy, when hepatic loading with iron occurs. In both approaches, supplemented sows showed an improved hematological status during pregnancy and after parturition. Supplemented sows also had better liver iron stores than unsupplemented control sows [70,71]. However, both the hematological and iron status of the piglets born to supplemented sows were similar to those of piglets born to untreated controls [70,71]. In contrast, Li et al. [72] observed an improvement in piglet RBC parameters compared to those of the unsupplemented control group, but at the same time, suspected that this improvement could be related to the consumption of maternal feces after birth, which is a typical behavior of piglets. Indeed, it has been found that the fecal iron content of iron-supplemented sows is very high [57]. Most studies reported a slightly improved iron status of piglets born to sows supplemented with iron, but iron supplementation did not affect the further development of the piglets [46]. The fact that iron supplementation in pregnant sows does not translate clearly to a higher iron status in their progeny strongly suggests that the placental transfer of iron to fetuses is insufficient, even in the presence of excess iron. However, the iron concentration in the placenta of iron-supplemented sows is higher than that in untreated animals. This finding may be explained by the increased expression of placental iron transporters [70], especially that of TfR1, which is responsible for iron uptake and also indicates increased iron uptake by placental tissue. At the same time, increased expression of Fpn, the only known iron exporter, is to be expected, yet no changes in the expression of this protein were observed. This finding may indicate iron retention in the placental tissue [57,70], which may be due to the increased expression of fetal hepcidin, the main inhibitor of Fpn. The fetal liver can produce hepcidin during embryonic development and thus may regulate placental iron transport and fetal iron homeostasis [73]. Such fetal hepcidin upregulation can indicate high fetal iron levels and is hypothesized to be a response to fetal iron overload [19,57]. However, one report noted that hepatic iron stores in piglets from supplemented sows were indeed elevated compared to controls [72]. Piglets born to iron-supplemented sows may receive adequate iron during fetal development, yet still show signs of developing IDA shortly after delivery. This phenomenon suggests that while iron supplementation during pregnancy is beneficial for both sows and piglets, it may still be inadequate to ensure sufficient iron stores in the early stages of the piglets’ postnatal life.

It should be noted that in all of the studies mentioned in this review, the scientific research was conducted on healthy sows that did not suffer from symptoms of anemia. Whether iron supplementation would benefit sows and piglets suffering from IDA requires further investigation in the field. 

### 3.4. The Impact of Iron Supplementation in Pregnant Sows on the Iron Content of Milk

After birth, the main dietary iron source for piglets is sow’s milk; however, this milk is not rich in iron (1.3–1.5 mg/L) [74], and its concentration is not sufficient to ensure proper levels of this microelement in developing piglets. Attempts have been made to establish if iron supplementation in sows during pregnancy or during lactation could influence the iron content in milk. Experimental data have shown that supplementation of sows can slightly improve milk iron content [52] or conversely, that supplementation has no effect [57]. Xing et al. [55] attempted to increase the iron level of milk by adding iron-saturated lactoferrin to it, in amounts ranging from 1.56 to 3.76 mg/L, but even this addition was not sufficient to prevent the development of IDA and decreased growth rates in piglets. 

**Table 1:** Studies documenting attempts to supplement pregnant sows to improve the iron status of mothers and their offspring. n.d., not defined; HGB, hemoglobin level; RBC, red blood cell level.

Author (Year)	Formulation	Dose of Fe	Length of Supplementation	Breed of Pigs	Effect on Piglets
McGowan (1924) [4]	Fe₂O₃	40 g per day	Last weeks of pregnancy	n.d.	HGB— 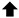 RBC— 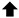 Liver Fe—n.d. Serum Fe—n.d.
Hart et al. (1930) [38]	Fe_2_(SO_4_)_3_ Fe₂O₃	10–250 mg per day	During pregnancy	Poland China, Duroc Jersey, and Chester White	HGB— 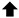 RBC— 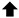 Liver Fe— 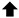 Serum Fe—n.d.
Pilgrim and Qureshi (1952) [59]	iron dextran	100 mg per day	During pregnancy	n.d.	HGB— 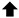 RBC—n.d. Liver Fe—n.d. Serum Fe—n.d.
Rydberg et al. (1959) [45]	iron dextran solution	1 g in a single dose	14 days before parturition	n.d.	HGB— 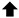 RBC— 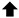 Liver Fe—n.d. Serum Fe—n.d.
Pond et al. (1961) [44]	iron dextran	1 g or 5 g in a single dose	From day 100 of pregnancy	Berkshire and Yorkshire	HGB—no change RBC— 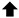 Liver Fe—n.d. Serum Fe—n.d.
Spruill et al. (1971) [48]	FeSO_4_	200 mg per day	94 days before prepartum	Hampshire and Yorkshire	HGB— 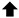 RBC—n.d. Liver Fe—n.d. Serum Fe—no change
Lillie and Frobish (1978) [49]	FeSO_4_	30 or 60 mg per day	During pregnancy	Duroc	HGB—no change RBC—n.d. Liver Fe—n.d. Serum Fe— 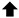
Ducsay et al. (1984) [47]	iron dextran	5.5 g (1.1 g per injection)	From day 40 to 60 of pregnancy	n.d.	HGB— 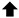 RBC— 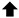 Liver Fe— 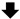 Serum Fe—n.d.
O’Connor et al. (1989) [69]	FeSO_4_x7H_2_O	200 mg per day	During pregnancy	n.d.	HGB— 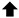 RBC—n.d. Liver Fe—n.d. Serum Fe—n.d.
Egeli et al. (1998) [75]	amino acid chelated iron or glutamic chelated iron	300 mg per day 650 mg per day	Last 3 weeks of pregnancy	Norwegian Landrace	HGB— 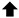 RBC— 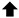 Liver Fe—n.d. Serum Fe—no change
Peters and Mahan (2008) [15]	chelated to soy proteinor FeSO_4_	20 mg per day	During pregnancy	Yorkshire Landrace	HGB— 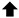 RBC—n.d. Liver Fe—n.d. Serum Fe—n.d.
Wang (2014) [52]	organic iron complex or FeSO_4_	650 mg per day	From day 84 of gestation	n.d.	HGB— 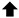 RBC— 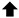 Liver Fe—n.d. Serum Fe— 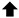
Zhao et al. (2015) [76]	chelated iron (bacterial iron)	n.d.	From day 84 of gestation	Yorkshire Landrace	HGB—no change RBC—n.d. Liver Fe—n.d. Serum Fe—no change
Jahan et al. (2017) [77]	lactoferrin	1 g per day	During pregnancy	Large White, Landrace, Duroc	HGB—n.d. RBC—n.d. Liver Fe—n.d. Serum Fe—n.d.
Buffler et al. (2017) [71]	Fe(II) SO_4_ 7H_2_O	688 mg per day, on average	During pregnancy	German Landrace	HGB— 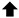 RBC—n.d. Liver Fe— 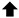 Serum Fe—no change
Li et al. (2018) [72]	Fe-Gly	150 mg 240 mg 330 mg 420 mg per day, on average	From day 86 of gestation	Landrace x Large White sows	HGB— 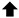 RBC— 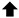 Liver Fe— 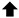 Serum Fe— 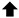
	FeSO_4_xH_2_O				HGB— 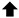 RBC— 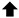 Liver Fe—n.d. Serum Fe — 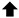
Wan et al. (2018) [70]	ferrous N-carbamylglycinate chelate or FeSO_4_	447 mg per day 457 mg per day	From day 86 of gestation	Landrace x Large Yorkshire	HGB—n.d. RBC—n.d. Liver Fe— 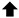 Serum Fe—n.d.
Barros et al. (2019) [78]	Yes Minerals Iron^®^	1.3 g per day	From day 84 of gestation	Topigs Norsvin^®^	HGB—n.d. RBC—n.d. Liver Fe—n.d. Serum Fe—n.d.
Mazgaj et al. (2020) [57]	sucrosomial ferric pyrophosphate	60 mg per day	From day 80 of gestation	990 line	HGB—no change RBC—no change Liver Fe—no change Serum Fe—no change
Zhang et al. (2022) [54]	lactoferrin and Fe-Gly	400 mg 700 mg 1 g per day	From day 80 of gestation	no info	HGB—n.d. RBC—n.d. Liver Fe—no change Serum Fe— 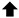
Xing et al. (2023) [55]	lactoferrin or heme iron or Fe-Gly	700 mg per day 280 mg per day 1.5 g per day	From day 33 of gestation	Landrace x Yorkshire	HGB—n.d. RBC—n.d. Liver Fe— 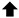 Serum Fe— 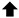

## 4. Discussion

Iron is an essential microelement required for various physiological functions in the body, including oxygen transport, energy metabolism, and DNA synthesis. Iron deficiency is a common problem in piglets and can lead to anemia and decreased growth rates [1]. Iron supplementation in pregnant sows has been suggested as a convenient procedure for improving piglets’ iron status. Iron supplementation of pregnant sows is desirable from a farming perspective, as this strategy for preventing IDA in piglets is considered to be more cost-efficient and less labor-intensive than parenterally supplementing piglets with iron dextran, and it avoids the stressful effects of that strategy. Yet, how to ensure adequate iron stores for piglets during pregnancy remains an unanswered question. Over the years, as outlined in this review, researchers and pig farmers have been trying different methods to increase the iron in sows and thus increase iron loading to the developing fetuses. Since the early 1980s and through the 1990s, farmers and researchers have struggled with the problem of IDA in high-performing pig breeds, which are developed and crossbred to produce high numbers of piglets per litter, each growing rapidly after birth [16]. One of the first methods addressing this problem was the use of injectable iron supplements into piglets shortly after birth [8]. However, this method was found to be labor-intensive, time-consuming, and stressful for piglets. That being said, it should be underlined that the intramuscular injection of FeDex is a cheap and effective procedure for preventing IDA in piglets.

Lately, efforts have centered around enhancing the iron reserves in pregnant sows by administering oral iron supplements, in the hope of facilitating improved iron transport to the fetuses and thus preventing IDA in newborn piglets altogether. Although the effectiveness of this method in improving iron levels in piglets is still under investigation, available data indicate that its effect is likely very limited and that it does not prevent the development of anemia in piglets to a satisfactory level. A very interesting aspect of the research is reexamining the placenta as a unique player in the iron transfer from the pregnant female to fetuses. This issue of placental iron transport is being raised in an increasing number of studies, yet most have been in mice and may not include differences in the architecture of the mouse and pig placentae [19,79]. 

## 5. Conclusions

IDA is a common nutritional disorder in suckling piglets, which can negatively impact their health, growth, and development. Iron supplementation of pregnant sows has been and is still considered to be an important strategy for preventing neonatal IDA in pigs. The rationale for supplementing pregnant sows is to increase their systemic iron status and consequently enhance iron transfer across the placenta, thus fortifying the iron stores in the fetuses; these stores then serve as an abundant source of endogenous iron during early postnatal life. Over the years, pregnant sows have been subjected to various methods, doses, formulations, and durations of iron supplementation, with only limited success. Further studies related to transplacental iron transport are needed to elucidate the limited ability of iron-supplemented sows to deliver this microelement to their fetuses.
